# Bridging repair of the abdominal wall in a rat experimental model. Comparison between uncoated and polyethylene oxide-coated equine pericardium meshes

**DOI:** 10.1038/s41598-020-63886-9

**Published:** 2020-04-24

**Authors:** Alessandro Pasculli, Angela Gurrado, Giuseppe Massimiliano De Luca, Antonietta Mele, Andrea Marzullo, Annarosa Mangone, Saverio Cellamare, Valentina Ferraro, Fatima Maqoud, Maria Cristina Caggiani, Francesco Rana, Giuseppe Cavallaro, Francesco Paolo Prete, Domenico Tricarico, Cosimo Damiano Altomare, Mario Testini

**Affiliations:** 1 University of Bari “A. Moro”, Department of Biomedical Sciences and Human Oncology, Unit of Academic General Surgery “V. Bonomo”, Bari, Italy; 2 University of Bari “A. Moro”, Department of Pharmacy-Drug Sciences, Bari, Italy; 3 University of Bari “A. Moro”, Department of Emergency and Organ Transplantation. Academic Unit of Pathology, Bari, Italy; 4 University of Bari “A. Moro”, Department of Chemistry, Bari, Italy; 5 Sapienza University of Rome. Department of Surgery “P. Valdoni”, Rome, Italy; 6 Present Address: University of Catania, Department of Biological, Geological and Environmental Sciences, Catania, Italy

**Keywords:** Preclinical research, Reconstruction

## Abstract

Biological meshes improve the outcome of incisional hernia repairs in infected fields but often lead to recurrence after bridging techniques. Sixty male Wistar rats undergoing the excision of an abdominal wall portion and bridging mesh repair were randomised in two groups: Group A (N = 30) using the uncoated equine pericardium mesh; Group B (N = 30) using the polyethylene oxide (PEO)-coated one. No deaths were observed during treatment. Shrinkage was significantly less common in A than in B (3% vs 53%, P < 0.001). Adhesions were the most common complication and resulted significantly higher after 90 days in B than in A (90% vs 30%, P < 0.01). Microscopic examination revealed significantly (P < 0.05) higher mesh integrity, fibrosis and calcification in B compared to A. The enzymatic degradation, as assessed with Raman spectroscopy and enzyme stability test, affected A more than B. The PEO-coated equine pericardium mesh showed higher resistance to biodegradation compared to the uncoated one. Understanding the changes of these prostheses in a surgical setting may help to optimize the PEO-coating in designing new biomaterials for the bridging repair of the abdominal wall.

## Introduction

Incisional hernia affects 10–20% of laparotomies and represents the most common long-term complication after abdominal surgery^1–3^. Since their first description by Usher *et al*. in 1985^4^, synthetic prostheses significantly reduced recurrence rate after the first incisional hernia repair, from more than 20% to less than 10%^5,6^. Nevertheless, they are commonly contraindicated in situations in which the exposure to any additional bacterial burden constitutes an excessive risk of surgical site infection^7,8^.

According to the Ventral Hernia Working Group (VHWG), incisional hernias which are at high risk of surgical site occurrences, because of comorbidities or contamination/infection of the operative field, should be treated using biological meshes; this should also be recommended in those cases in which the complete rectus closure is not possible and bridging technique is unavoidable^9^. Although some controversies arose about the influence of technique in determining the surgical site occurrences^10^, and other authors designed and validated other scores^11,12^, VHWG recommendations appear to be still the most reliable guidelines available in the literature. Unfortunately, despite a study protocol published in 2013 by Mariette *et al*., a randomised controlled trial designed to provide a high level of evidence on the use of biological meshes in such contexts is still lacking^13^.

Many different biological and biosynthetic meshes are available for ventral hernia repair, with various features, such as cross-linking or different coating methods to adjust their degradation to the abdominal wall repair necessities, but integration and reabsorption of these materials still need more extensive studies and reliable measurements^14^. On the other hand, the advantages of using biological and biosynthetic meshes have been rigorously questioned in a recent review by Köckerling *et al*., especially in case of bridging techniques, because of the very high recurrence rate (up to 80%)^15^. On the basis of these literature findings, the use of biological and biosynthetic meshes is no longer considered routinely recommended.

The pericardium tissue is widely used in the production of biological prostheses, abdominal wall meshes, vascular grafts, heart valves, and so on. The pericardial tissue derives from different species, such as bovine and porcine^16^, ostrich^17^, kangaroo^18^, and equine^19^. The first experimental report about the use of the equine pericardium dates back to about forty years ago^20^. Since then, it has been used mainly in cardiothoracic and vascular surgery^21,22^, but also in neurosurgery^23,24^, orthopaedics^25^ and plastic surgery^26,27^. A literature survey did not reveal studies devoted to the use of equine pericardium in the abdominal wall repair. Moreover, albeit the growing interest in the molecular analysis of abdominal wall reconstruction devices^28–31^, no standardised methods are still available for assessing in detail the biochemical changes affecting collagen of the biological meshes once implanted into a living organism. In this regard, the advantages and limitations of Raman spectroscopy, in the clinical setting, have been recently reviewed^32^.

In this study, an equine pericardium prosthesis, already commercialised for tissue repair applications in plastic surgery, orthopaedics and neurosurgery, was implanted as substitute of the abdominal wall in a rat model and compared for its post-surgical outcomes to a new equine pericardium patch coated with polyethylene oxide (PEO). This study aims at investigating if and how much PEO coating on equine pericardium mesh used as a substitute of the abdominal wall in a rat model can effectively slow down its degradation, possibly opening the route to design and preparation of new biomaterials usable to repair large defects of the abdominal wall, being at the same time resistant to infection and recurrence. The equine pericardium prostheses were studied for 90 days through a multidisciplinary approach, by synergistically combining ultrasonographic, autoptic, histological, spectroscopic, biochemical and physicochemical techniques.

## Results

### Ultrasound and macroscopic examination

**Figure 1:**
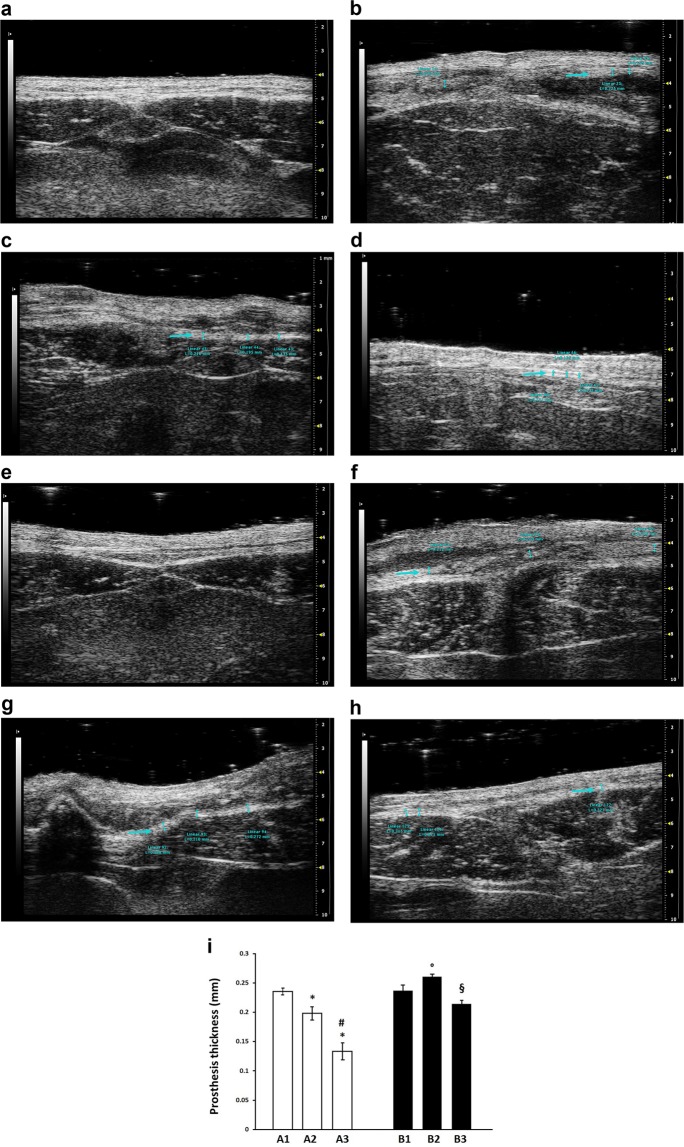
(**a**–**d**) Ultrasonography sample images of B-mode acquisition of a Group A rat abdomen at the level of the surgical scar. (**a**) before the implant. (**b**) 30 days after the implant (A1). (**c**) 60 days after the implant (A2). (**d**) 90 days after the implant (A3). The blue arrows identify the mesh. (**e**–**h**) Ultrasonography sample image of B-mode acquisition of the Group B rat abdomen at the level of the surgical scar. (**e**) before the implant. (**f**) 30 days after the implant (B1). (**g**) 60 days after the implant (B2). (**h**) 90 days after the implant (B3). The blue arrows identify the mesh. (**i**) Prosthesis thickness measured by ultrasound in each experimental group. Each bar is the mean ± standard deviation from five to nine measurements. Statistical analysis by unpaired Student’s *t*-test indicates P < 0.05 versus A1 group (*), P < 0.01 versus A2 group (#), P < 0.05 versus B1 group (°) and P = 0.001 versus B2 group (§).

Figure 1(a–d,i) shows the comparison of the ultrasound measured thickness of the prosthetic bridge for the different subgroups employing the uncoated mesh. A significant thickness reduction of 15.7% and 43.4% was observed in A2 compared to A1 (P = 0.04), and in A3 compared to A1 (P < 0.001), respectively. A significant (P = 0.006) thickness reduction of 32.9% was also observed in A3 vs A2.

Figure 1(e–i) shows the ultrasound measured thickness of the prosthetic bridge for the different subgroups employing the PEO-coated mesh. A significant (P = 0.035) thickness increase of 9.9% was observed in B2 vs B1; a decrease of 9.5% was observed in B3 vs B1 (P = 0.07). A significant (P = 0.001) thickness decrease of 17.7% was observed in B3 vs B2.

When comparing Group B vs A, a significant difference was observed in B2 vs A2 (P = 0.0001) and in B3 vs A3 (P = 0.0003).

No rat died during the treatment. Table 1 shows the autoptic findings. Overall morbidity was 58%, with no significant differences between Group A and B. The shrinkage was significantly (P < 0.001) more common in Group B than A and also (P < 0.05) in subgroups B1 and B2 compared with A1 and A2, respectively. However, displacement of the mesh was never observed. Incisional hernias were more common in Group A vs B, although this difference was not statistically significant.

**Table 1:** Autoptic examination results.

N (%)	Group A (N = 30)				Group B (N = 30)				P*			
	Total	A1 (N = 10)	A2 (N = 10)	A3 (N = 10)	Total	B1 (N = 10)	B2 (N = 10)	B3 (N = 10)	A vs B	A1 vs B1	A2 vs B2	A3 vs B3
Shrinkage	1 (3)	0 (0)	1 (10)	0 (0)	16 (53)	5 (50)	7 (70)	4 (40)	**0.000**	**0.033**	**0.020**	0.087
Displacement	0	0	0	0	0	0	0	0	na	na	na	na
Wound dehiscence	1 (3)	1 (10)	0	0	4 (13)	4 (40)	0	0	0.353	0.303	na	na
Wound infection	0	0	0	0	2 (7)	2 (20)	0	0	0.492	0.474	na	na
Rejection	0	0	0	0	2 (7)	1 (10)	1 (10)	0	0.492	1.000	1.000	na
Incisional hernia	3 (10)	0	0	3 (10)	0	0	0	0	0.237	na	na	0.211
Seroma	1 (3)	0	0	1 (10)	0	0	0	0	1.000	na	na	1.000
Periprosthetic abscess/collection	1 (3)	1 (10)	0	0	3 (10)	2 (20)	1 (10)	0	0.612	1.000	1.000	na
Bowel obstruction	0	0	0	0	1 (3)	0	0	1 (10)	1.000	na	na	1.000
Bowel perforation	0	0	0	0	1 (3)	0	0	1 (10)	1.000	na	na	1.000
Haemorrhage/haematoma	0	0	0	0	0	0	0	0	na	na	na	na
Viscero-parietal adhesions	7 (23)	1 (10)	3 (30)	3 (30)	12 (40)	2 (20)	1 (10)	9 (90)	0.267	1.000	0.582	**0.0062**
Viscero-visceral adhesions	1 (3)	0	0	1 (10)	1 (3)	0	0	1 (10)	1.000	na	na	1.000
Entero-cutaneous or entero-enteric fistulas	0	0	0	0	0	0	0	0	na	na	na	na
Nil^a^	16 (53)	7 (70)	6 (60)	3 (30)	9 (30)	5 (50)	3 (30)	1 (10)	0.067	0.650	0.370	0.582

Data given as absolute value and percentage in brackets. Group A: non-coated prosthesis; Group B: coated prosthesis. A1 and B1: evaluation after 30 days; A2 and B2: evaluation after 60 days; A3 and B3: evaluation after 90 days.

*Fischer’s exact test.

^a^Rats without complications.

Moreover, the development of viscero-parietal adhesions after 90 days from implant was significantly (P < 0.01) higher in B3 than in A3. When detected, these adhesions found in both groups were constantly single or double, directed from the small intestine to the center of the exposed mesh.

### Microscopic examination

**Figure 2:**
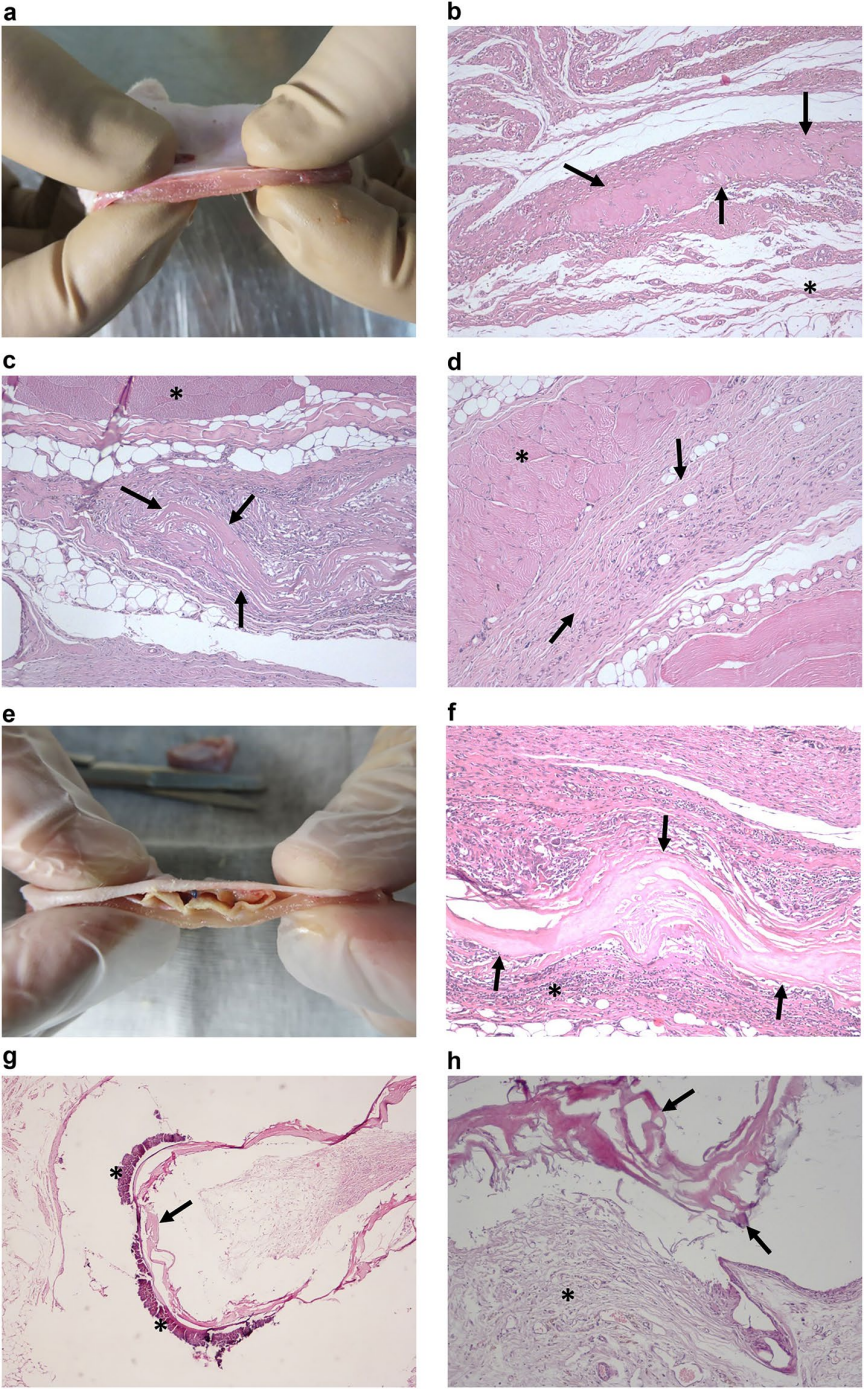
(**a**–**d**) Group A: (**a**) Group A1 prosthesis soon after its removal from the rat. (**b**) Group A1 10x hematoxylin-eosin stained section, the arrows show a fragment of the prosthesis, the asterisk identifies subcutaneous tissue. (**c**) Group A2 10x hematoxylin-eosin stained section, the arrows indicate a fragment of the prosthesis, the asterisk identifies the muscular layer. (**d**) Group A3 10x hematoxylin-eosin stained section, the prosthesis is no longer recognisable and is substituted by fibrous tissue (arrows), the asterisk identifies the muscular layer. (**e**–**h**) Group B: (**e**) Group B1 prosthesis soon after its removal from the rat. (**f**) Group B1 10x hematoxylin-eosin stained section, intact prosthesis (arrows) encircled by inflammatory infiltrate (asterisk). (**g**) Group B2 4x hematoxylin-eosin stained section, calcification (asterisks) on the edge of the prosthesis (arrow). (**h**) Group B3 10x hematoxylin-eosin stained section, the prosthesis is still visible (arrow) and appears to be encircled by fibrous tissue (asterisk).

Figure 2 shows the macroscopic aspect of the two prostheses as soon as removed from rats and the main histological characteristics. The pathological analysis, as shown in Table 2, demonstrated that integrity of the prosthesis and calcification were significantly (P < 0.05) higher in Group B as compared with Group A, and also in the comparison between each subgroup.

**Table 2:** Microscopic examination results.

N (%)	Group A (N = 30)				Group B (N = 30)				P*			
	Total	A1 (N = 10)	A2 (N = 10)	A3 (N = 10)	Total	B1 (N = 10)	B2 (N = 10)	B3 (N = 10)	A vs B	A1 vs B1	A2 vs B2	A3 vs B3
Prosthesis integrity	1 (0–2)	1 (1–2)	1 (0–1)	0 (0–0)	2 (1–2)	2 (2–2)	2 (1–2)	1.5 (1–2)	**<0.0001**	**0.0001**	**0.0001**	**<0.0001**
Inflammatory response	1 (0–1)	1 (0–1)	1 (0–1)	1 (0–1)	1 (0–2)	1 (0–2)	1 (0–1)	1 (0–2)	0.101	0.6901	1.000	**0.0383**
Fibrosis	0 (0–1)	0 (0–0)	0 (0–0)	1 (0–1)	0 (0–2)	0 (0–1)	0 (0–1)	1 (1–2)	**0.0265**	0.1462	0.1462	**0.0214**
Neoangiogenesis	0 (0–1)	0 (0–0)	1 (0–1)	0.5 (0–1)	0 (0–1)	0 (0–1)	0 (0–0)	1 (0–1)	1.000	0.1462	**0.0043**	0.0571
Calcification	0 (0–0)	0 (0–0)	0 (0–0)	0 (0–0)	1 (0–2)	1 (0–1)	1 (0–2)	0.5 (0–1)	**<0.0001**	**0.0043**	**0.0018**	**0.0118**

Data given as median and range. Group A: non-coated prosthesis; group B: coated prosthesis. A1 and B1: evaluation after 30 days; A2 and B2: evaluation after 60 days; A3 and B3: evaluation after 90 days.

*Wilcoxon rank-sum test.

Concerning inflammatory response and fibrosis, they resulted significantly lower (P < 0.05) in A3 than in B3. Fibrosis was also significantly (P < 0.05) higher in the whole Group B compared to A. Neoangiogenesis resulted significantly higher (P < 0.005) in A2 than in B2. Supplementary Figures S1–S6 show large size pictures concerning histological features.

### Raman spectroscopy

The spectral positions of all the found bands are listed in Supplementary Table S1, that includes the positions of the signals revealed on one side of the prosthesis in Group B, which have been observed to appear invariably white and crusted (called B1w, B2w, B3w). A large part of the signals found both on A0 and B0 can be assigned to collagen spectrum, characterised by the peptide backbone bands of amide I (1560–1725 cm^−1^) and amide III (1200–1350 cm^−1^)^33–39^, in detail, the type I collagen features are confirmed^34^.

**Figure 3:**
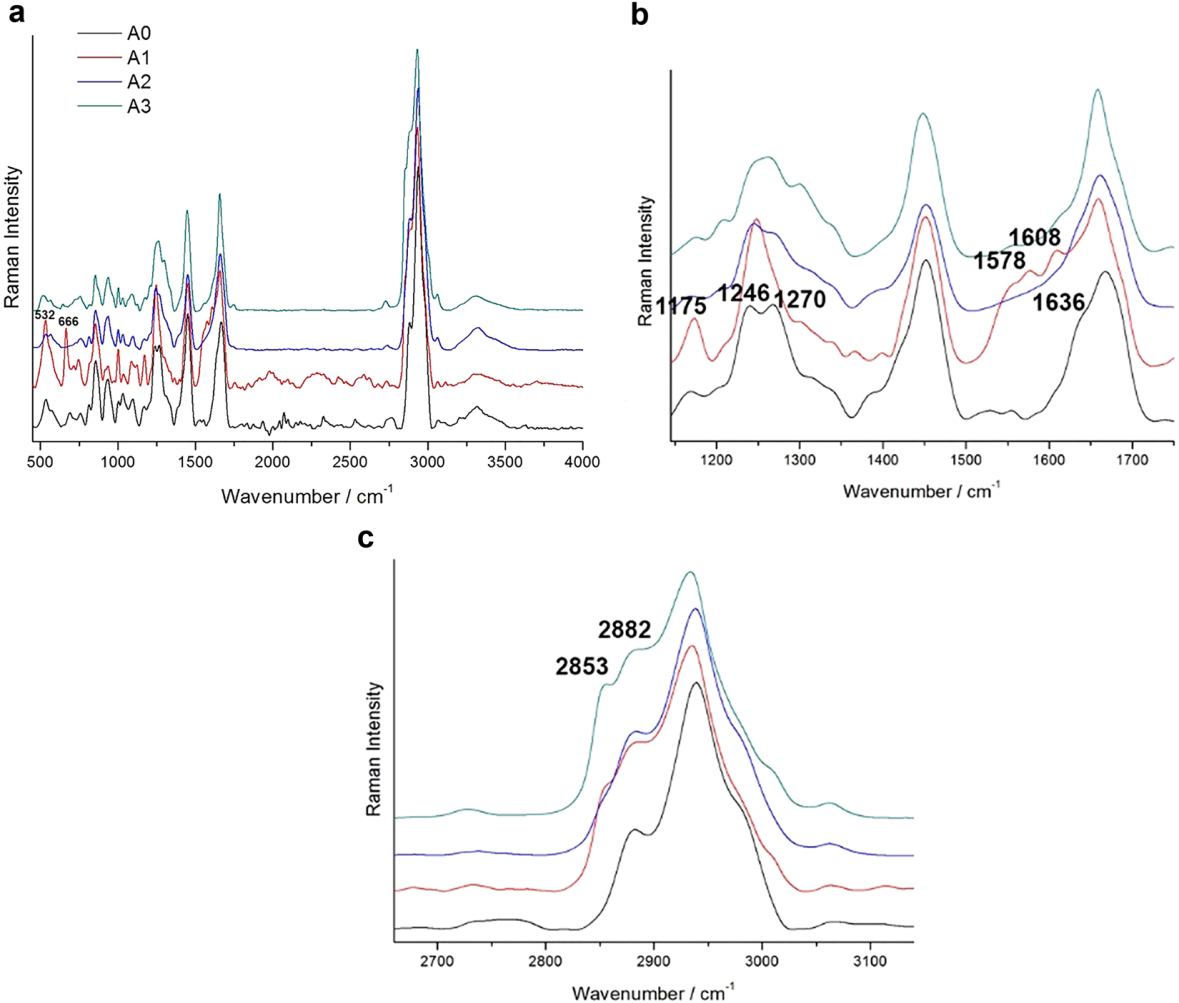
Representative baseline-subtracted Raman spectra of Group A prostheses before and after surgery in the 500–4000 cm^−1^ region (**a**) and details of the 1100–1800 (**b**) and 2600–3200 cm^−1^ (**c**) regions. The intensities are normalised to the band at 2940 cm^−1^, the spectra are shifted on the Y-axis for better readability. The peaks cited in the text are marked.

**Figure 4:**
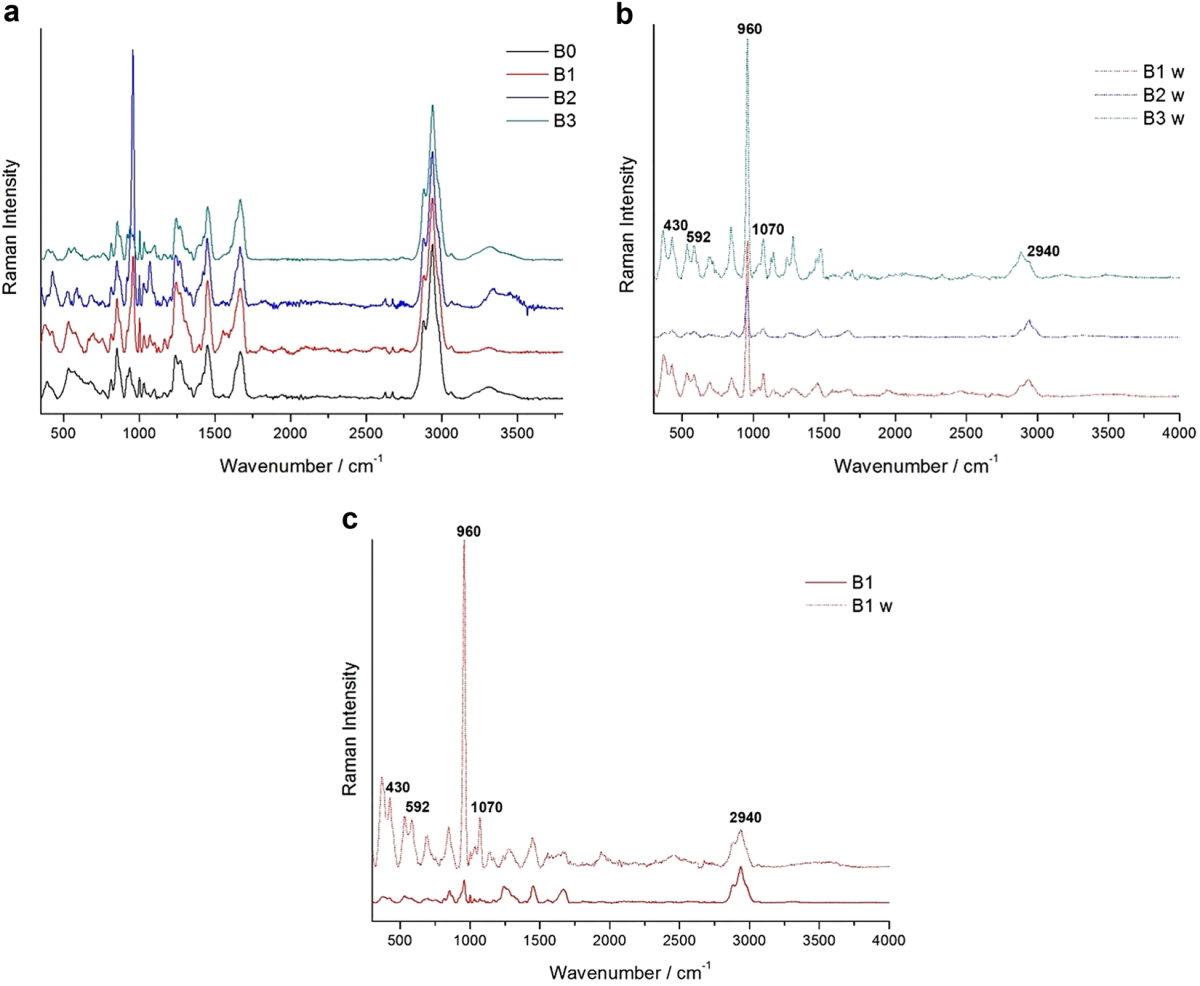
Representative baseline-subtracted Raman spectra of Group B prostheses before and after surgery (**a**), after surgery on the white side (**b**), and comparison between B1 on the two sides (**c**). For each single group of spectra, the intensities are normalised to the band at 2940 cm^−1^, the spectra are shifted on the Y-axis for better readability. The peaks cited in the text are marked.

Representative spectra of all the phases are reported in Fig. 3 for Group A and Fig. 4 for Group B. Concerning Group A, difficulties arose in the Raman acquisition due to fluorescence both in A0 and in A1, which appeared to be the most affected spectrum: the peak at 532 cm^−1^ was more intense than in the other spectra, while that at 666 cm^−1^ was present only in this case; both signals are linked to sulphur, the former to S-S^34^ and the latter to C-S cystine^39^ stretching vibrations. As for the region between 1100 and 1800 cm^−1^ (Fig. 3b), in the after-implant spectra, the intensity ratio between the peaks at ca. 1246 cm^−1^ linked to proline-rich regions, and that at ca. 1270 cm^−1^ linked to proline-poor regions, resulted varied concerning the as-such prosthesis, especially in the A1 spectrum, where the 1270 cm^−1^ peak reached its minimum reflecting a maximum in the proline residues amount^34^; this ratio is instead inverted in the A3 sample.

Both in A1 and A3, the component at 1636 cm^−1^ of the broad band at ca. 1660 cm^−1^, linked to C=C amide I stretching^35,37,39^, disappeared, while in A1, other components related to phenylalanine and tyrosine (1175, 1608 cm^−1^)^33,35,37^, and to proline and hydroxyproline (1578 cm^−1^)^35,37^, increased. Furthermore, in the CH_3_, CH_2_ stretching band (Fig. 3c), in all the after-implant samples, the components at 2882 and 2853 cm^−1^ were increased.

Concerning the Group B prostheses, the collagen spectrum generally prevailed (Fig. 4a). The I_1270_/I_1246_ ratio remained constant, and no change was observed for the CH_3_, CH_2_ stretching band. On the other hand, the B spectra were characterized by the appearance of the signals of phosphate (PO_4_^3−^) at 430, 592, 960, 1070 and 1076 cm^−1^ ^35,36,39^, and carbonate (CO_3_^2−^) at 1070 cm^−1^ ^35,36^. A weak band at 960 cm^−1^ was already present on the B0 prosthesis, but the peak notably increased in the after-implant ones, accompanied by the appearance of the other cited bands (Fig. 4b). On the white and crusted side of the after-surgery B prostheses, the intensity ratio between the main phosphate band at 960 cm^−1^ and the 2940 cm^−1^ one always resulted higher than the other side (Fig. 4c).

### Enzymatic stability assessed by mass spectrometry

At the end of the incubation time in the digest solution (24 h), a nearly complete dissolution of the prosthesis of Group A was observed by visual inspection. Instead, the sample of the prosthesis of Group B remained almost unaltered.

**Figure 5:**
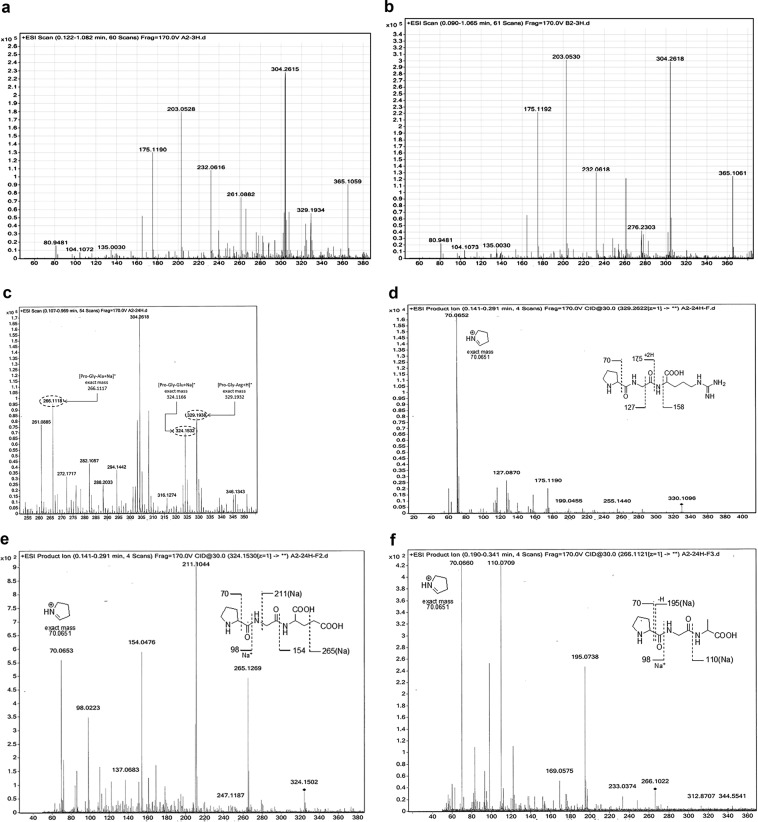
HRMS and tandem MS/MS fragmentation traces of representative supernatant samples of prostheses at different times of incubation in digest solutions at 37 °C: (**a**) MS spectrum up to 390 m/z (ESI scan recorded up to 1200 m/z, positive mode) of a representative Group A prosthesis sample after 3 h incubation; (**b**) MS spectrum up to 390 m/z (ESI scan recorded up to 1200 m/z, positive mode) of a representative Group B prosthesis after 3 h incubation; (**c**) putative structure assessment of some m/z signals of a representative Group A sample after 24 h incubation; tentative assignments by MS/MS fragmentation analysis of the signals at (**d**) m/z 329 (Pro-Gly-Arg), (**e**) m/z 324 signal (Pro-Gly-Glu) and (**f**) m/z 266 signal (Pro-Gly-Ala).

The high-resolution mass spectrometry (HRMS) of the supernatant digest solutions of the uncoated prostheses at 3 h-incubation showed a signal at m/z 329 (Fig. 5a), which was not detectable in the PEO-coated mesh samples (Fig. 5b). After 6 h (and for the whole 24 h-incubation time), the HRMS spectrum of the PEO-coated mesh samples remained almost unchanged, whilst the signal at m/z 329 of the uncoated prosthesis samples increased (not shown). After 24 h-incubation of the uncoated prostheses of Group A (and not those of Group B), the HRMS spectra of the supernatant digest solutions, compared with the MS spectra at 3 h, showed a doubled intensity of the signal at m/z 329 and several further signals (Fig. 5c). Tandem mass spectrometry (MS/MS) experiments reasonably suggested to assign the signals at m/z 329 (Fig. 5d), 324 (Fig. 5e) and 266 (Fig. 5f) to the tripeptides sequences Pro-Gly-Arg, Pro-Gly-Glu and Pro-Gly-Ala, respectively.

## Discussion

Large incisional hernias in which the complete rectus closure is impossible, which fall into grade 3–4 patients according to VHWG classification^9^, often represent a real surgical challenge. Biological mesh repair, also accomplished by bridging technique, has been suggested in such cases^9^, despite controversies related to the very high recurrence rate^15^. Many biological and biosynthetic materials have been already studied as replacement of the abdominal wall^15^. Although routinely used in cardiothoracic and vascular surgery, neurosurgery, plastic surgery and orthopaedics^20–27,40,41^, equine pericardium has never been employed in bridging repair of the abdominal wall, perhaps because of described complications in other fields of employment likely related to host tissue reactions leading to degradation and leak^24^ or fibrosis and shrinkage^41^.

In this study, a new prosthesis made of equine pericardium coated with PEO (Group B) was studied as a substitute of the abdominal wall in a rat model and compared to the uncoated one (Group A). A multidisciplinary comparative study was carried out 30, 60 or 90 days (subgroups A1 and B1, A2 and B2, A3 and B3, respectively) after surgical implantation of the meshes, through *in vivo* (ultrasonographic, US) and *ex vivo* (autoptic, microscopic examination, Raman spectroscopy) analyses. As far as we can infer from the literature, the use of ultrasound techniques in measuring the thickness of the implanted prosthesis in rats is unprecedented, as well as the synergistic use of Raman spectroscopy and mass spectrometry in understanding enzymatic stability and molecular transformations of these biological materials in a clinical setting.

US showed a significant thickness reduction of the prosthetic bridge in Group A during the time, whereas, in contrast, the average thickness of the PEO-coated prostheses in Group B at first appeared to increase and then decreased significantly after 90 days of implantation. The described trend reveals a faster and constant degradation of the uncoated prosthesis and an increased resistance to reabsorption of the PEO-coated one.

Autoptic findings revealed in the prostheses of Group B a propensity to adhesions and shrinkage significantly higher than in Group A. However, higher incidence of recurrence was observed in Group A. These results are consistent with the hypothesis that PEO coating, conferring to the equine pericardium resistance to biodegradation, reduces the occurrence of incisional hernias, but on the other hand increases the risk of adhesions, as already proved in case of non-absorbable materials implanted into peritoneal cavity^42^. In Group B a liability to septic complications was observed, although no significant differences appeared in comparison with Group A. At time of autoptic exploration, prostheses of Group B were always macroscopically well identifiable, whereas those of Group A were difficult to isolate, even in A1 subgroup.

Microscopic examination confirmed the slower degradation rate of the PEO-coated mesh. The prostheses of Group B often resulted intact and with calcium deposits. Inflammation was higher in Group B, probably because of shear stress caused by the rigid prosthesis on the surrounding muscular, peritoneal or subcutaneous tissues. As a consequence, fibrosis was higher in Group B compared to Group A.

Raman examination demonstrated that the two prostheses had different spectra. Many of the differences found in Group A between the prostheses before and after implant, were not remarkable in Group B. Indeed, in the uncoated mesh group, there were time-dependent differences in the samples, such as the changes in the proline-rich/proline-poor regions and in the CH_3_, CH_2_ stretching band. In the PEO-coated mesh group, instead, the signals of phosphate and carbonate characterized the spectra.

The ultrasound, histological and Raman spectra differences between Groups A and B prompted us to compare the *in vitro* enzymatic stability of the uncoated vs the PEO-coated prosthesis. At the end of the incubation time in the digest solution (24 h), a nearly complete dissolution of the uncoated prosthesis was observed by visual inspection. In contrast, the PEO-coated mesh sample remained almost unaltered. This observation can be considered in itself a proof of the very different stability of the two types of biomaterials. It can be reasonably inferred that the PEO coating does not allow the enzyme pool to attack the protein matrix in 24 h at 37 °C.

The amino acid sequence of the three type I collagen α chains polypeptides consist of Xaa-Yaa-Gly triplet repeats, where Xaa or Yaa can be any amino acid^43^, that are often (2 S)-proline (Pro), and (2S,4R)-4-hydroxyproline (Hyp). Pro-Hyp-Gly is the most common triplet in collagen, easily detectable by MS. Previous studies about enzymatic stability of collagen showed different amino acid composition. In 1994, Todhunter *et al*., investigating equine type I and type II collagens obtained from skin and flexor tendon and articular cartilage^44^, reported that hydroxylation of proline accounts for about 40% in both types, while hydroxylation of lysine account for 23% to 34% from tendon (type I), and about 50% from cartilage (type II); in 2004, Angele *et al*., reported that the amount of hydroxylysine and lysine in equine collagen resulted higher than bovine collagen which had a slightly higher amount of proline and hydroxyproline residues; collagenase digestion was able to completely degrade both samples within 1.5 h without significant inter-species differences^45^. In this study, the change over time of enzymatic digestion was checked by Q-TOF-MS analysis to detect, in the supernatant samples, peptide markers of the proteolytic cleavage of native collagen (Fig. 5c–f).

The coating is a process that requires the association in a hydrogel of PEO and another polymer, that is hydroxy-propyl-methylcellulose, in a pH 7.2 phosphate-buffered saline containing also ascorbic acid as visco-elasticity regulator. The coated mesh is not cross-linked but the hydrogel undergoes a cross-linking during the sterilization process by beta radiation. This is probably the cause of the lengthening of the degradation time, leading to a foreign body-like response with the formation of a fibrotic capsule and consequent increase in the risk of adhesions.

Notwithstanding the unsatisfactory data about recurrences after bridging repair with biological mesh^15^, we believe that further investigation about these materials must be undertaken. This study underlines how the behavior of a biomaterial can be dramatically modeled by the processing with a polymeric coating. Recent reviews^46–48^, claim that an ideal material for abdominal wall reinforcement should be able to be integrated and slowly degraded while providing a matrix to native tissue growth. The ideal mesh should also show low encapsulation phenomena, low adhesiogenic potential, resistance to infections and capacity of remesothelialization. It seems that the uncoated equine pericardium mesh is too prone to early degradation, whereas on the opposite the coated one appears too prone to the encapsulation. Our data suggest that the biomaterials for the repair of the abdominal wall should be hybrid between biologic and synthetic meshes.

Taken together, the results of this study show that the PEO coating of equine pericardium prosthesis, although exposing to adhesive and infectious phenomena, could be useful in modulating its resistance to biodegradation when implanted in rats in bridging repair surgical technique, overall reducing the early incisional hernia incidence. This feature may reduce the incidence of recurrence when these biomaterials completely replace the abdominal wall. While further studies are required to optimize the biophysical properties of the coated equine pericardium prostheses, this work suggests that a multidisciplinary protocol, synergistically combining US techniques with microscopy, MS and Raman spectroscopy, can be effective in assessing the biomaterial properties in abdominal ventral hernia bridging repair in further animal models. In this context, the Raman spectroscopy data reported herein can represent a good starting point for next research aimed at optimizing the technique and the proportion of PEO coating in equine pericardium, and ultimately at designing biomaterials useful in bridging repair of abdominal wall.

In extrapolating these results to humans, this study shows some inherent limitations. Indeed, equine pericardium prosthesis average thickness (between 0.2 and 0.4 mm), appropriate for rats’ abdominal wall, would not be adequate to cure humans’ abdominal wall defects. Moreover, upright posture confers to the abdominal muscles in humans a different static and dynamic function that rats cannot adequately simulate. Nevertheless, the rat model herein presented allowed us to study, from diverse points of view and with different tools, the changes of the implanted biomaterial to better understand the biophysical behavior of uncoated and coated equine pericardium prostheses in a surgical setting.

## Methods

### Animals and ethics statement

Between March and July 2016, 60 male rats (Wistar, aged 6.5 ± 2.6 months; weight 487.3 ± 89.2 g), were sourced from an independent vendor (Charles River, Wilmington, MA, USA). The animal care and treatments were carried out according to the Guide for the Care and Use of Laboratory Animals, 8^th^ edition (National Research Council (US) Committee for the Update of the Guide for the Care and Use of Laboratory Animals, Washington (DC): National Academies Press (US); 2011) and under the supervision of the local veterinary official (prof. Angelo Quaranta). The study protocol was approved by the Italian Ministry of Health (Rome, n° 725/2025-PR, July 17^th^, 2015) and experiments performed at the Department of Pharmacy-Drug Sciences of the University of Bari, following authorisation protocol n° 12608, May 23^rd^, 2016, by Organismo Preposto al Benessere Animale (O.B.P.A.). Vendor health reports indicated that the animals were free of known pathogens.

In the pre-experimental period, the rats were hosted in standard conditions (temperature: 22 ± 1 °C; relative humidity: 50 ± 5%; 12 hours light/dark alternating cycle; standard laboratory diet and water ad libitum). The animals were fasted for 24 hours before the surgical procedure. All surgery was performed under an aseptic setting and general anaesthesia, and all efforts were made to minimise suffering. The general anaesthesia was induced by the intraperitoneal injection of Zoletil 50/50 mg/ml (tiletamine hydrochloride and zolazepam hydrochloride, Pfizer, New York, NY, USA) using refracted doses for a maximum dose of 40/40 mg/kg (average 11.1 ± 2.3 mg), and the inhalation of isoflurane at an induction concentration of 3.5%. The isoflurane maintained general anaesthesia during ultrasound examination, at a level of 1.5–2%. The combination of these drugs induced a rapid anaesthetic action, complete muscle relaxation, without arrhythmias, epilepsy, hepatic/renal toxicity, nor spastic movements during awakening^49^. Analgesic (buprenorphine, 15 ± 3 µg) and antibiotic (enrofloxacin, 10 mg) single doses were administered by subcutaneous or intramuscular injection. Anaesthetised rats were shaved on the surgical site, placed on a heated surgical pad in dorsal recumbence, disinfected with povidone-iodine and draped with sterile gauze^49^.

After one post-operative day of water-only diet, the free oral standard diet was administered to the operated animals. Additional doses of buprenorphine and enrofloxacin were delivered during the first two post-operative days. Bodyweight, temperature, motility, bowel movements, diuresis, alimentation and status of the wound were monitored by two blinded pharmacologists, experienced in trials on laboratory animals, once per day during the first post-operative week, then once a week for the remaining study period. No additional analgesic nor antibiotic doses were used after the second post-operative day. During the first two postoperative days, a reduction in body weight not higher than 10% was observed, together with impaired motility in either group. Within ten days from the surgical procedure, all rats fully recovered their weight, locomotor activity and general behaviour.

At the end of the ultrasound examination (30, 60, or 90 days after the surgical procedure), the experimental animals were sacrificed under saturated CO_2_ atmosphere.

### Surgical procedure

The sixty rats underwent the surgical removal of a portion of their abdominal wall and the implant of a prosthesis according to a randomisation in two groups: Group A (N = 30) received the uncoated 50 × 50 mm single layer (0.393 ± 0.01 mm thick) equine pericardium patch (HRT-20; Bioteck, Arcugnano (VI), Italy); Group B (N = 30) received the coated 50 × 50 mm single layer (0.221 ± 0.12 mm thick) prosthesis (HRT-HMW-20; Bioteck, Arcugnano (VI), Italy). The two groups were sub-randomized in 3 sub-groups with ten rats each, based on the time of post-operative ultrasound examination, sacrifice and abdominal wall excision, at 30 (subgroups A1 and B1), 60 (subgroups A2 and B2) and 90 (subgroups A3 and B3) days after the implant. The randomisation was performed assigning rats to random codes, each of them corresponding to a type of prosthesis and a post-operative time.

Two blinded surgeons, experienced in the abdominal wall surgery, performed in both groups a median laparotomy extended from the xiphoidal process till the sub-umbilical region, with the detachment of subcutaneous tissue from the aponeurosis of the abdominal muscles from both sides of the wound. A portion of the abdominal wall (muscular layers and serous membrane) was excised from both sides to create a 1.5 cm wide defect, in the midpoint of the wound. The prosthesis was shaped and implanted as a substitute of abdominal wall, in a subcutaneous position, overlapping for about 1 cm on all sides, secured with 5–7 polypropylene stitches of 2/0–3/0 (Assut Europe, Rome, Italy). The skin was then closed over the prosthesis.

### Ultrasound and macroscopic examination

Two blinded pharmacologists, experienced in ultrasound examination in laboratory animals, used an ultrasonic biomedical microscanning system (Vevo 2100; VisualSonics, Toronto, ON, Canada) during general anaesthesia. The abdominal wall was shaved, and an ultrasound gel was used during image acquisition. The MS550 transducer (VisualSonics, Toronto, ON, Canada), working at the frequency of 40 MHz with an axial and lateral resolution of 40 µm and 90 µm respectively, was used to scan the surgical scar, from 0.5 cm above to 0.5 cm below in a transverse position, from 0.5 cm on the right to 0.5 cm on the left side in a longitudinal position, to be sure of recording images of the area in which the prosthesis substituted the abdominal wall. A B-mode acquisition was performed to measure the thickness of the prosthetic bridge between the abdominal muscular layers, as an average of three measurements. The ultrasound examination was used to identify shrinkage or displacement of the prosthesis.

Soon after their sacrifice, the rats underwent the full-thickness excision of the abdominal wall, including the prosthesis. During this procedure an abdominal autoptic examination was performed by the same surgeons that performed the prosthesis implant, verifying the following complications: wound dehiscence/infection, rejection, seroma, periprosthetic abscess/collection, incisional hernia, bowel obstruction/perforation, viscero-parietal/viscero-visceral adhesions, entero-cutaneous/entero-enteric fistulas.

### Preparation of histological specimens and microscopic examination

All the abdominal wall specimens were fixed for 24 h at room temperature in neutral buffered 10% formalin. Subsequently, slides 4 mm thick were obtained by cutting the fragments orthogonally as respect to the cutaneous surface, to achieve transverse sections including skin, subcutaneous tissue, muscular layer and, if present, the serous membrane. Such sections were taken making attention to avoid the polypropylene stitches and, where macroscopically recognisable, to include the prosthesis. The samples were embedded in paraffin, and thin serial sections 4 µm thick were cut and stained with Hematoxylin-Eosin. Finally, two blinded pathologists observed the sections at microscope.

The following histological parameters were evaluated: the integrity of the prosthesis, the grade of inflammation, fibrosis, neoangiogenesis, and calcification. To each item was assigned a semiquantitative score with a graded scale ranging from 0 (absence) to 2 (intense) through a mild-moderate stage scored as 1. As for the integrity of the prosthesis, the scale was graded as follows: structure microscopically intact (score 0), fragmented (score 1) or unrecognisable (score 2). Neoangiogenesis was scored as 0 in cases in which the formation of new vessels was absent; 1 where few vessels were visible and, finally, the score 2 was used for vascular crowding.

### Raman spectroscopy

A specimen of the removed prostheses and of the as-such ones (called A0 and B0) was used for Raman spectroscopy. The micro-Raman analyses were performed through a LabRAMHR Evolution (Horiba, Kyoto, Japan) spectrometer, equipped with a Peltier-cooled charge-coupled device detector (CCD), Ar+ 514 nm and 488 nm and He-Ne 633 nm lasers, coupled with a BH2 microscope (Olympus Corporation, Tokyo, Japan) provided with different objectives. The used parameters were adjusted after several tests finding a compromise between intensity, noise reduction and performance duration, making sure that the samples did not alter under the laser beam. The better results were obtained with the 633 nm laser, using a 50× long-working-distance (N.A. = 0.50) objective (Olympus Corporation, Tokyo, Japan) with a power of illumination on the sample of about 0.5 mW. The spectral resolution achieved with the 1800 g/mm grating was of about ±1 cm^−1^. The number of spots analysed was calibrated according to the dimensions of the samples, to collect a representative set of results for each one of them. A linear baseline was subtracted from the raw spectra and intensities were normalized to the band at 2940 cm^−1^, using the software LabSpec6 (Horiba, Kyoto, Japan). Both the as-such prostheses in Group A and B and those removed after 30, 60 and 90 days were analysed without preparation.

### Enzymatic stability and mass spectrometry

Nutrient Mixture F-10 Ham (sodium bicarbonate, without l-glutamine, liquid, sterile-filtered) at a final concentration of 43%, collagenase (from *Clostridium Histolyticum* Sigma C_9697) at a final concentration of 0.7 mg/ml, trypsin-EDTA (ethylenediaminetetraacetic acid) (Sigma T_4299) at a final concentration of 0.5×, HEPES (4-(2-hydroxyethyl)-1-piperazineethanesulfonic acid) buffer (Sigma H_3375), at a final concentration of 1%, and Fetal Bovine Serum (Sigma F_6178), at an ultimate level of 5%, were used as received (all from Sigma-Aldrich, Merck KGaA, Darmstadt, Germany).

Ultraviolet-sterilized rectangular sections (1 × 0.5 cm, dry weight 0.03 to 0.035 g), were cut out from as-such prostheses of both groups, and placed into a 6-well plate; 1.5 ml of newly prepared digest solution was added in each well, and the plate was placed in a non-humidified incubator at 37 °C for 3 h, 6 h and 24 h. Three wells were prepared with the only digestive solution, without any prosthesis, to evaluate the effect on parameters under investigation after each incubation time. No significant efforts were made to control the activity of the enzyme because complete digestion was the only requirement of this essay. At the end of the incubation time, the supernatants were transferred in clean tubes and stored at −80 °C until mass analysis.

Quadrupole Time-of-Flight (Q-TOF) MS was performed on an electrospray ionisation (ESI) source and an Agilent-6530 (Agilent, Santa Clara, CA, USA) Q-TOF mass spectrometer. Positive and negative ion modes were used in mass detection. The source parameters were set as follows: drying gas flow rate: 11 l/min; gas temperature: 350 °C; pressure of nebuliser gas: 45 psig; Vcap: 4000 V in positive mode and 3000 V in negative mode; fragmentor: 120 V; skimmer: 45 V; and scan range: m/z 50–1200. The MS/MS analysis was acquired in targeted MS/MS mode with the collision energy ranging from 10 V to 40 V. The infusion rate was set at 10 µl/min.

Each tube containing the supernatants was defrosted and filtered by 0.22 µm polytetrafluoroethylene septa. The resulting solutions were injected into the ESI source. The MS spectrum of the digestive solution, without any prosthesis, was used as a reference blank (Supplementary Fig. S7).

### Statistical analysis

Comparisons were made using unpaired Student’s *t*-test for the thickness, which was assumed to be normally distributed; frequencies were compared using the Fisher’s exact test; scores were compared using Wilcoxon rank-sum test. A two-tailed *P*-value < 0.05 was considered statistically significant. There were two experimental groups (group A and group B): the calculation of the sample size was carried out considering Δ in terms of the variation of the shrinkage between the two groups equal to 0.5 through a statistical hypothesis based on literature data. The power of the study for the calculation of the sample was 0.95 in input and remained high in output (G * Power 3.1.9.2 and R i386 3.2.2) using a priori test of the power analysis. Statistical analyses were performed using Stata14 software (StataCorp LSJ, College Station, TX, USA).

## Supplementary information

Supplementary information.
