# Supplementary material for: Bridging repair of the abdominal wall in a rat experimental model. Comparison between uncoated and polyethylene oxide-coated equine pericardium meshes
Source: Sci Rep. 2020 Apr 24;10:6959. doi: 10.1038/s41598-020-63886-9 (PMC7181852; doi:10.1038/s41598-020-63886-9)
Supplement: Supplementary file 1 — Supplementary information. [file 41598_2020_63886_MOESM1_ESM.pdf]

Bridging repair of the abdominal wall in a rat experimental model. Comparison between uncoated and polyethylene oxide-coated equine pericardium meshes.

Alessandro Pasculli, Angela Gurrado, Giuseppe Massimiliano De Luca, Antonietta Mele, Andrea Marzullo, Annarosa Mangone, Saverio Cellamare, Valentina Ferraro, Fatima Maqoud, Maria Cristina Caggiani, Francesco Rana, Giuseppe Cavallaro, Francesco Paolo Prete, Domenico Tricarico, Cosimo Damiano Altomare, Mario Testini

Supplementary material:

Supplementary Table S1

Supplementary Figures S1-S7

Supplementary Table S1 – Raman lines of the samples analysed.

Group A				Group B				Groub B (crusted side)			Assignment	References
A0	A1	A2	A3	B0	B1	B2	B3	B1 w	B2 w	B3 w		
					426	*430*		*427*	*432*	*429*	$\nu_2(PO_4)^{-3}$	[36, 37]
530	532	531	531	531	532	533	535	532	537	533	ν(S-S)	[34]
570		571	567	570			570				Trp?	[40]
			579			583		582	580	580	_	
							594	*592*		*592*	$\nu_4(PO_4)^{-3}$	[36]
											$\nu_2(PO_4)^{-3}$	[37]
602		599	600	600							_	
620		621	621	620	620		621				Phe	[38]
		644	644				645				τ(CC) Tyr, Phe	[40]
	666				665			665			ν(C-S) cystine	[40]
694		696	696		698		698				_	
720		724	724	721			724			721	$\delta(CH_2)$	[33]
756	758	758	758	758			761				Trp?	[35, 40]
814	814	814	818	815	817	816	817				ν(CC)	[40]
											ν(CC), $\delta(CO_2)$ proteinbackbone	[39]
831			830								Pro, Hyp, Tyr	[40]
								848	847	846	_	
855	854	854	854	854	855	854	857			857	ν(CC) Pro, δ(CCH) Tyr	[33-36, 38-40]
874	873	873	873	873	875	872	875			876	ν(CC) Hyp	[33-36]
											ν(CC), δ(CCH) Hyp	[39]
920	920	920	920	920	920	920	921				ν(CC) Pro ring	[34, 36, 38-40]
938	938	938	938	939	938		939				ν(CC) α helix	[33, 34, 36, 38-40]
				960	*960*	*960*	*959*	*960*	*960*	*960*	$\nu_1(PO_4)^{-3}$	[36, 37]
968			968								Amide III	[38]
1002	1004	1004	1004	1003	1004	1002	1004	1004	1004	1003	ν_s(CC) ring breathing Phe	[33, 36-40]
1031	1033vw	1033	1033	1031	1033	1030	1033		1031	1030	δ(CH) Phe	[34, 35]

											o.p. $\delta(OH)$	
1043		1045	1045	1045	1043	1043	1047			1043	carboxyl	[38]
											Pro	[40]
1054	1050										_	
1062		1062	1062	1061	1060	1061	1062				$\delta(COC)$	[33]
					1070	*1071*		*1071*	*1072*	*1071*	$\nu_1(CO_3)^{-2}$, $\nu_3(PO_4)^{-3}$	[36, 37]
					1076						$\nu_3(PO_4)^{-3}$	[36, 40]
1082	1080		1085		1080		1080				$\delta(CN)/(CC)$	[33]
1100		1097		1100	1100	1098	1100			1102	$\nu_{as}(COC)$	[33]
1126	1125	1125	1125	1126	1126	1125	1126		1127	1126	$\nu(COC)$, $\nu(CN)$	[33, 35]
				1142	1145	1142				1143	_	
1164	1163	1163	1163	1163	1165	1161	1163			1164	Tyr	[40]
1175	1175	1175	1175	1175	1175	1175	1175		1176		$\nu(CC)$ Tyr	[33, 38]
1206	1206	1206	1206	1205	1205	1205	1205		1205		$\omega(CH_2)$ Tyr, Hyp	[36, 38, 40]
1236				1237		1238			1236	1236	Amide III $\nu(CN)$ $\delta(NH)$	[33, 36-39]
1246	1248	1244	1248	1244	1244	1247	1244				Amide III $\nu(CN)$ $\delta(NH)$	[33, 36-39]
1269	1270	1269	1266	1269	1271	1270	1271		1270		Amide III $\nu(CN)$ $\delta(NH)$	[36, 38, 39]
1278	1278	1278		1277		1280				1281	Amide III	[33, 36, 40]
	1300		1299								$\tau(CH_2)$, ω(CH3, CH2) / lipids	[40]
		1317	1317	1317		1316	1317				$\tau(CH_3, CH_2)$	[38, 40]
1341	1341	1340	1340	1344	1344	1345	1344				$\omega(CH_3, CH_2)$	[33, 38]
1366	1366	1366									Trp?	[40]
1376	1378	1378		1378	1378		1378				δ(CH3) lipids	[40]
1385			1388		1386	1386	1386				(CH_3)?	[40]
1395	1395	1395	1395	1398	1398		1398				$\delta(CH_2)$	[33, 40]
1423	1425	1425	1425	1423	1422	1422	1425		1422		$\nu(COO^-)$	[33, 38]
1450	1448	1448	1448	1451	1449	1450	1452	1448	1450	1448	δ(CH3, CH2)	[35, 37, 38]
1460				1464	1463	1464	1463				δ(CH3, CH2)	[38]

									1479	1477	_	
1556	1556	1556	1556	1558	1556						ν(CC) Trp?	[35, 40]
1585	1578	1584	1584								ν(CCH) aromatic Pro, Hyp	[36, 38]
1606	1608	1606	1606	1606	1607		1607				δ(CC)Phe, Tyr	[36, 38]
1639		1636		1640	1640	1640	1640		1635		ν(C=C) Amide I	[36, 38, 40]
1668	1658	1658	1658	1668	1663	1666	1667		1663		ν(C=O) Amide I	[33, 36-39]
			1684				1680				Amide I	[40]
										1695		
			1746								ν(C=O) lipids	[40]
		2734	2729				2735				_	
	2853	2853	2853								ν(CH_3, CH_2)	[40]
2881	2884	2882	2882	2880	2882	2880	2883	2885	2880	2880	ν(CH_3, CH_2)	[33, 37, 40]
2939	2936	2938	2933	2940	2940	2940	2941	2940	2938	2940	ν(CH_3, CH_2)	[33, 37, 40]
2980	2979	2979		2979	2978	2982	2983		2978		ν(CH_3, CH_2)	[33, 37, 40]
	3008		3006								ν_{as} (=CH) lipids	[40]
3066	3062	3062	3062	3063	3062	3063	3063	3064			ν(C=CH)	[33]
3210		3209	3209				3208				_	
3318	3318	3318	3318	3318	3318	3318	3318		3315		ν_s(NH)	[33]

All data are reported as cm^{-1}. Group A: non-coated prosthesis; Group B: coated prosthesis. A1 and B1: evaluation after 30 days; A2 and B2: evaluation after 60 days; A3 and B3: evaluation after 90 days.

Phosphate and carbonate positions are reported in italics. ν: stretching, νs: symmetric stretching, νas: antisymmetric stretching, δ: bending, τ: twisting, ω: wagging, o.p.: out of plane, Trp: tryptophan, Phe: phenylalanine, Tyr: tyrosine, Pro: proline, Hyp: hydroxyproline.

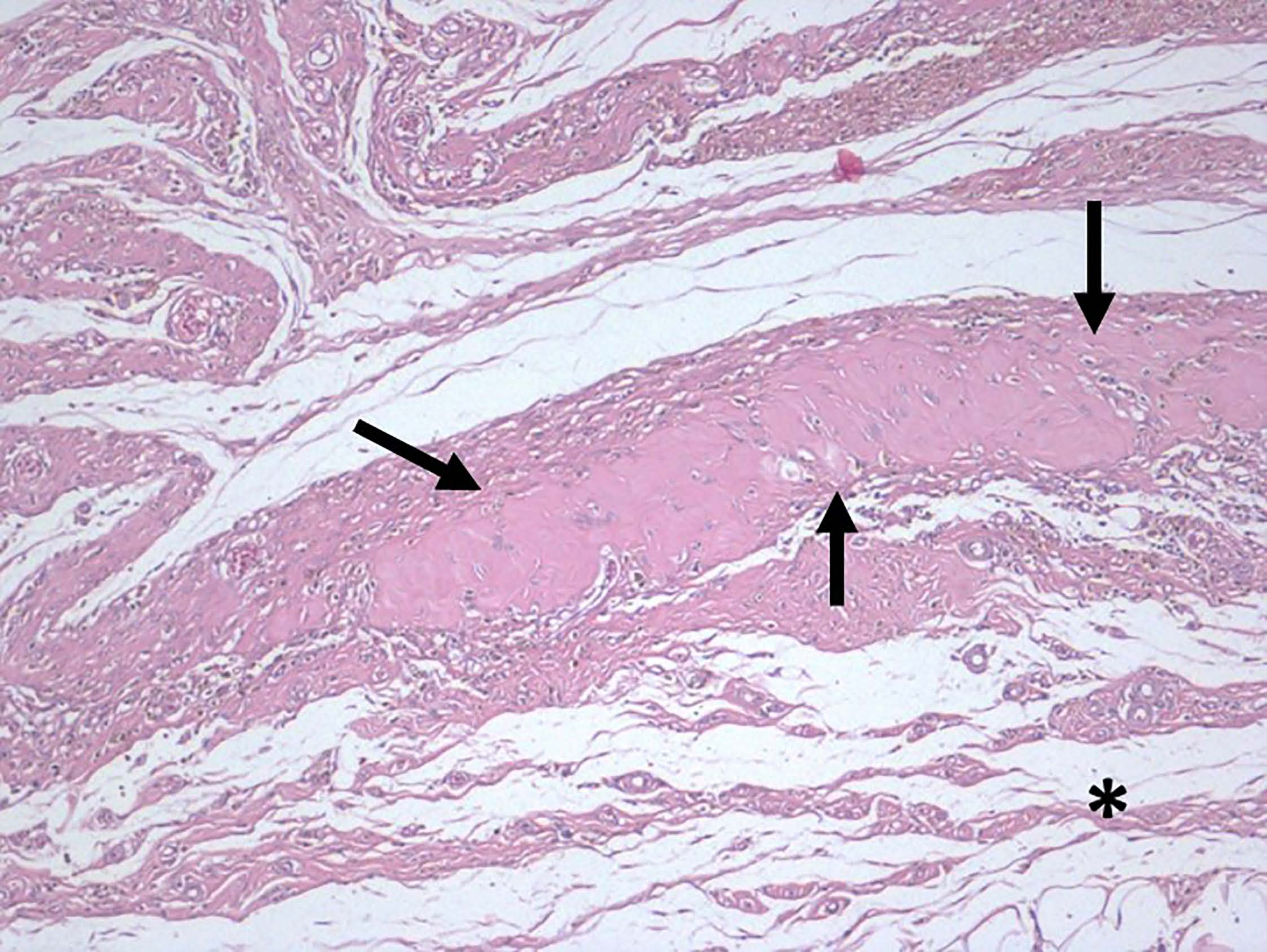

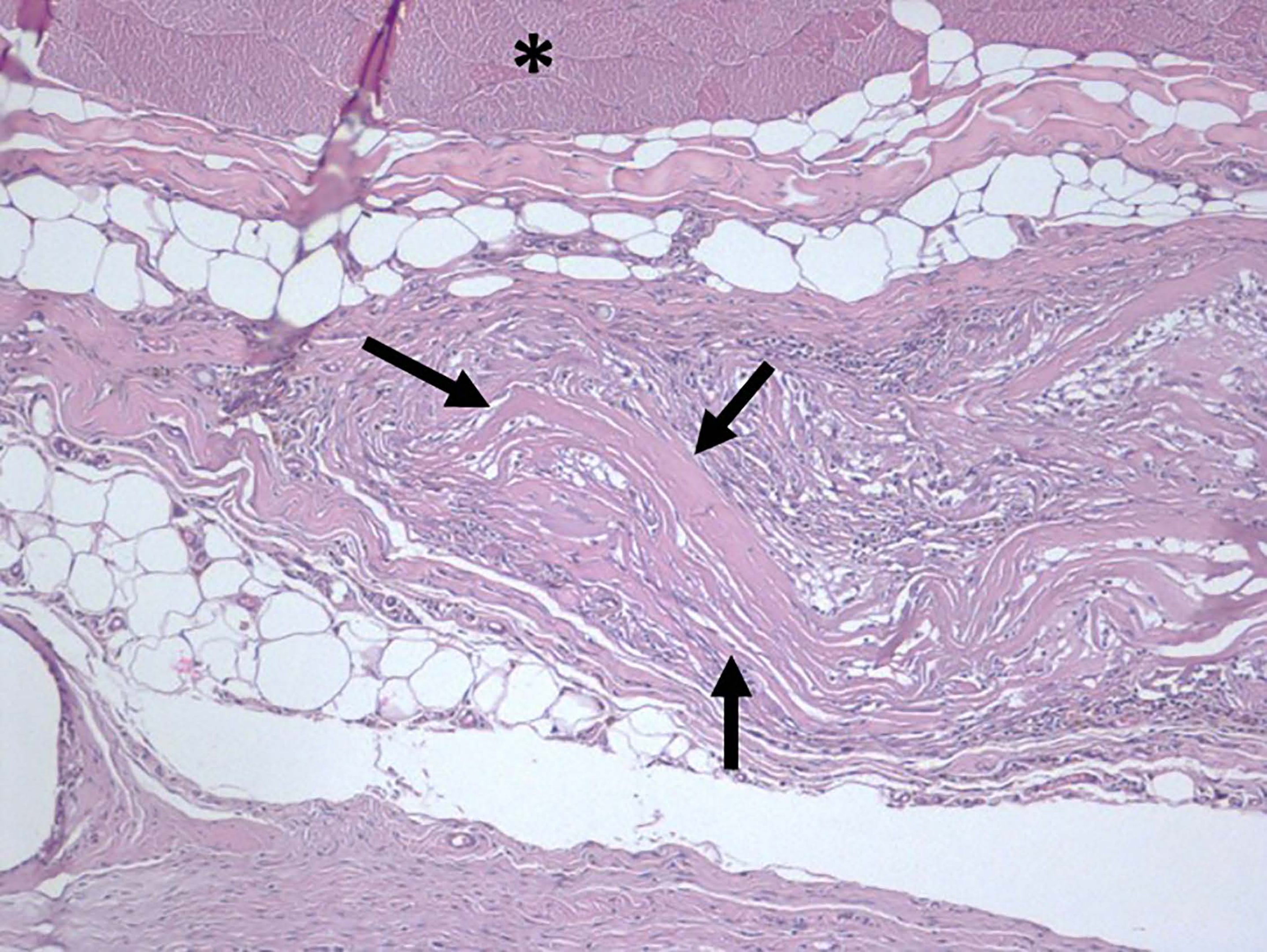

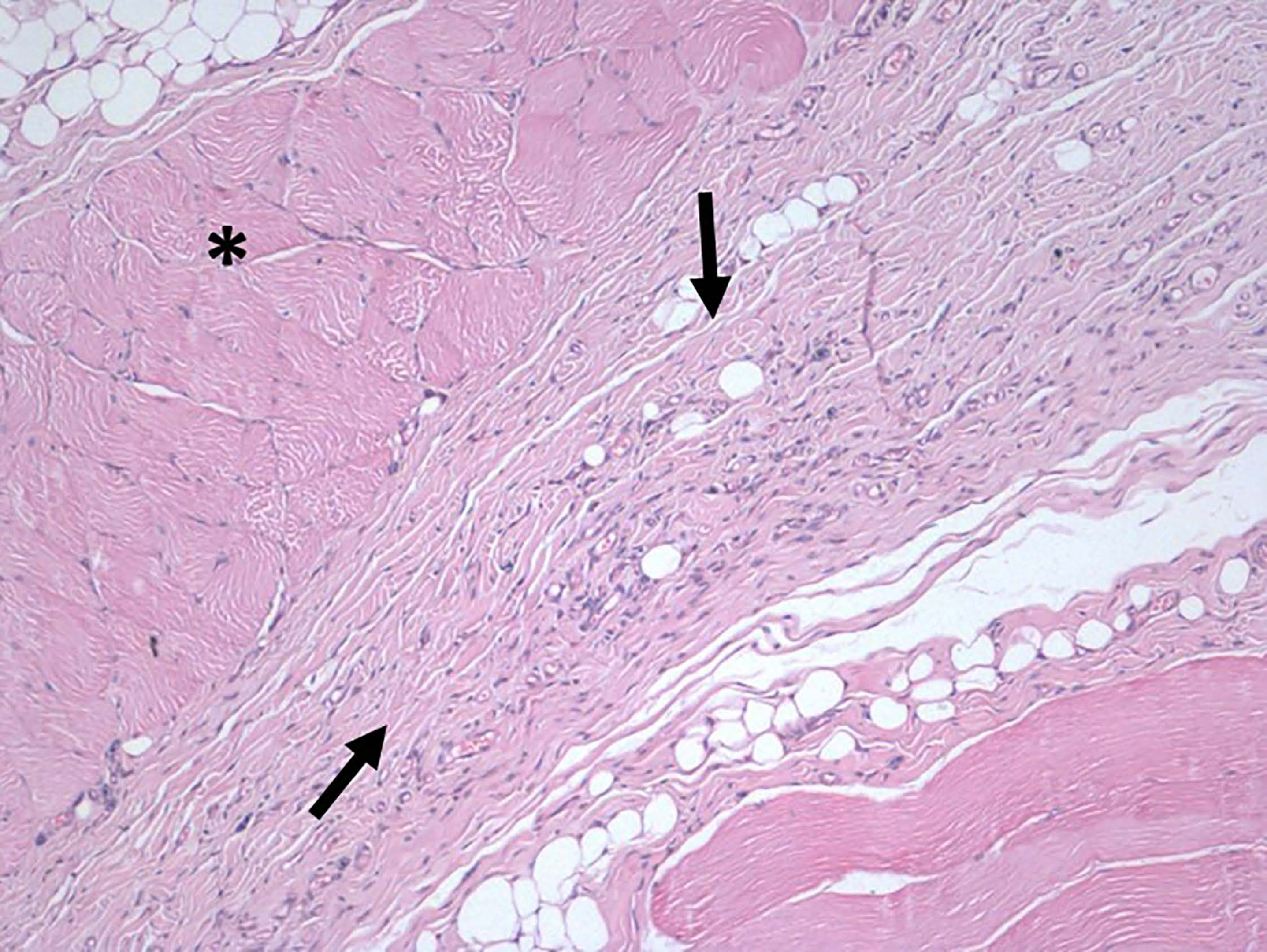

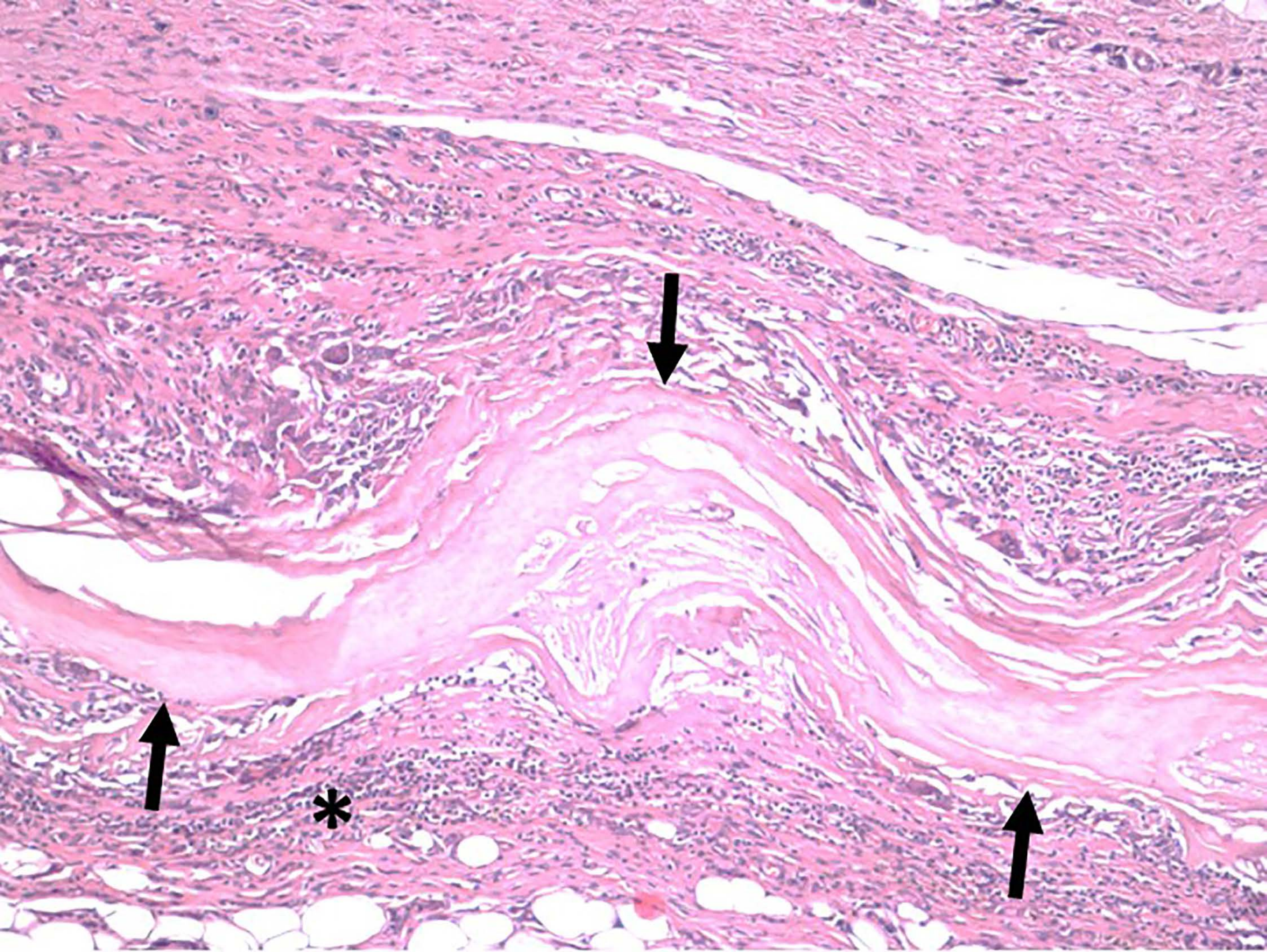

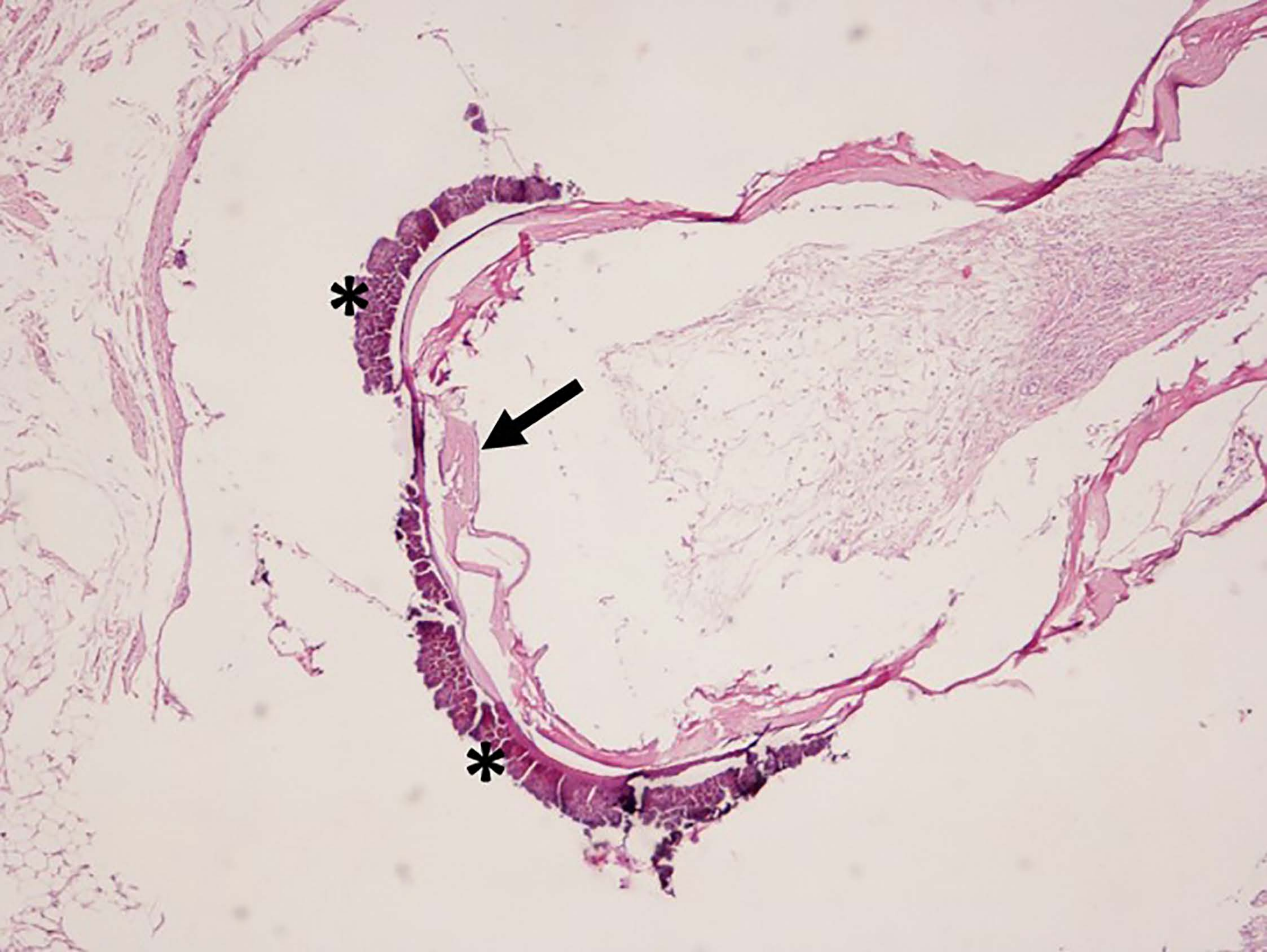

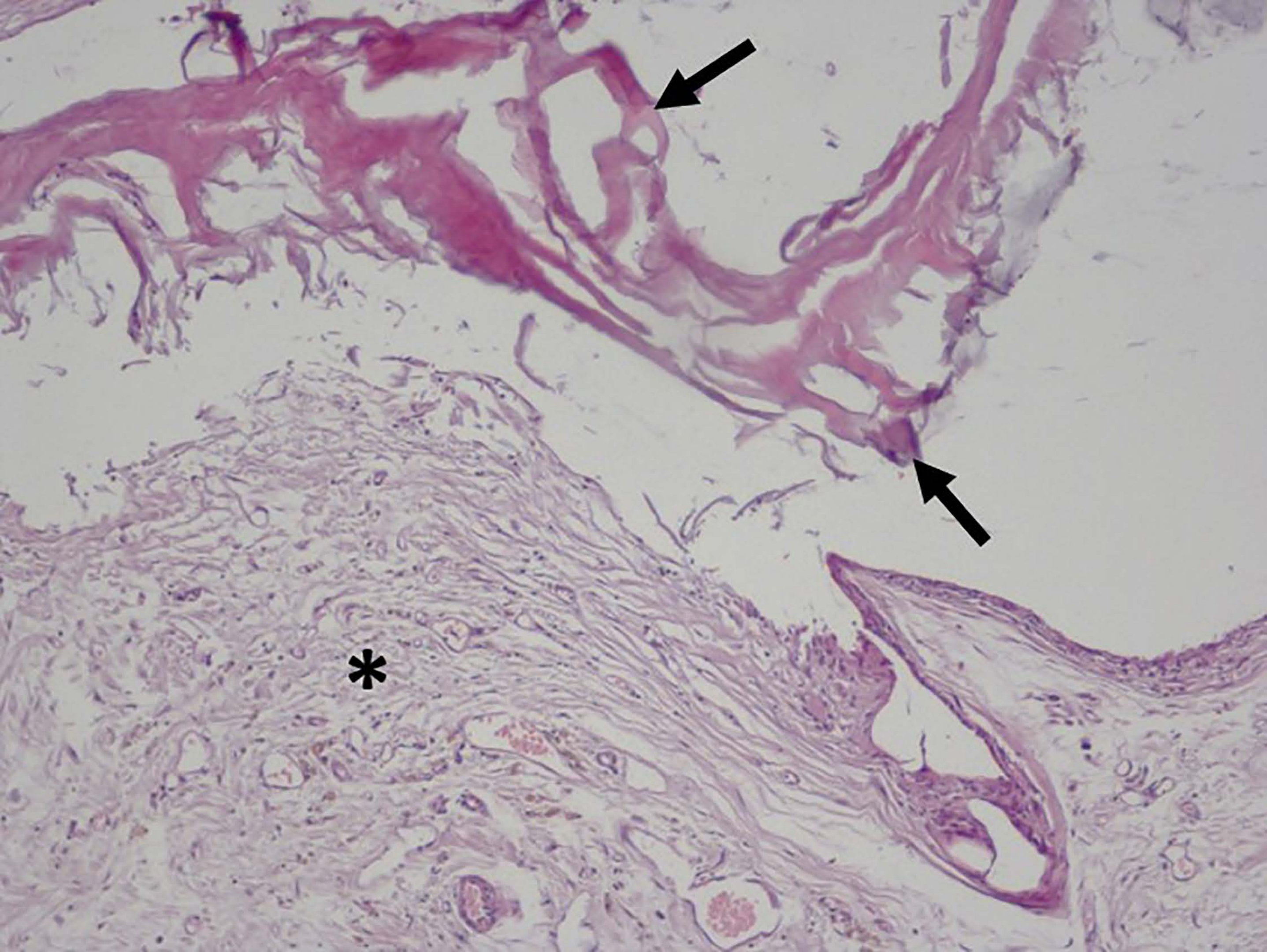

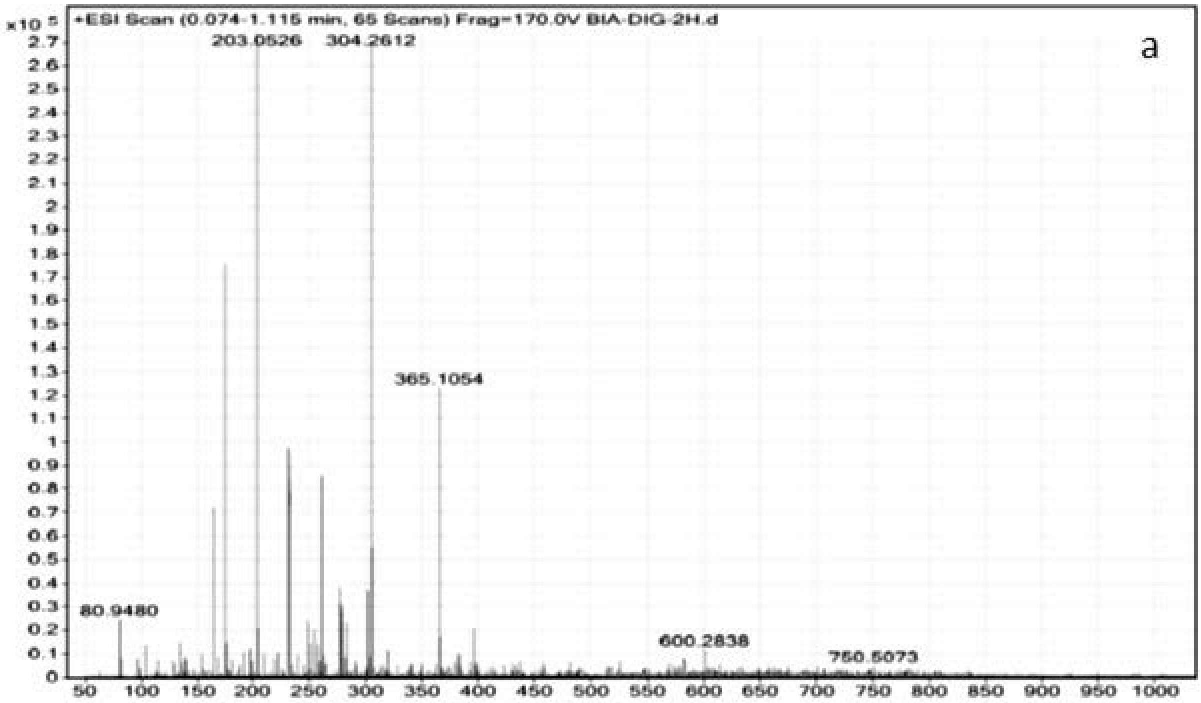
+ESI Scan (0.074-1.115 min, 65 Scans) Frag=170.0V BIA-DIG-2H.d
x10 5
a
203.0526
304.2612
365.1054
80.9480
600.2838
750.5073

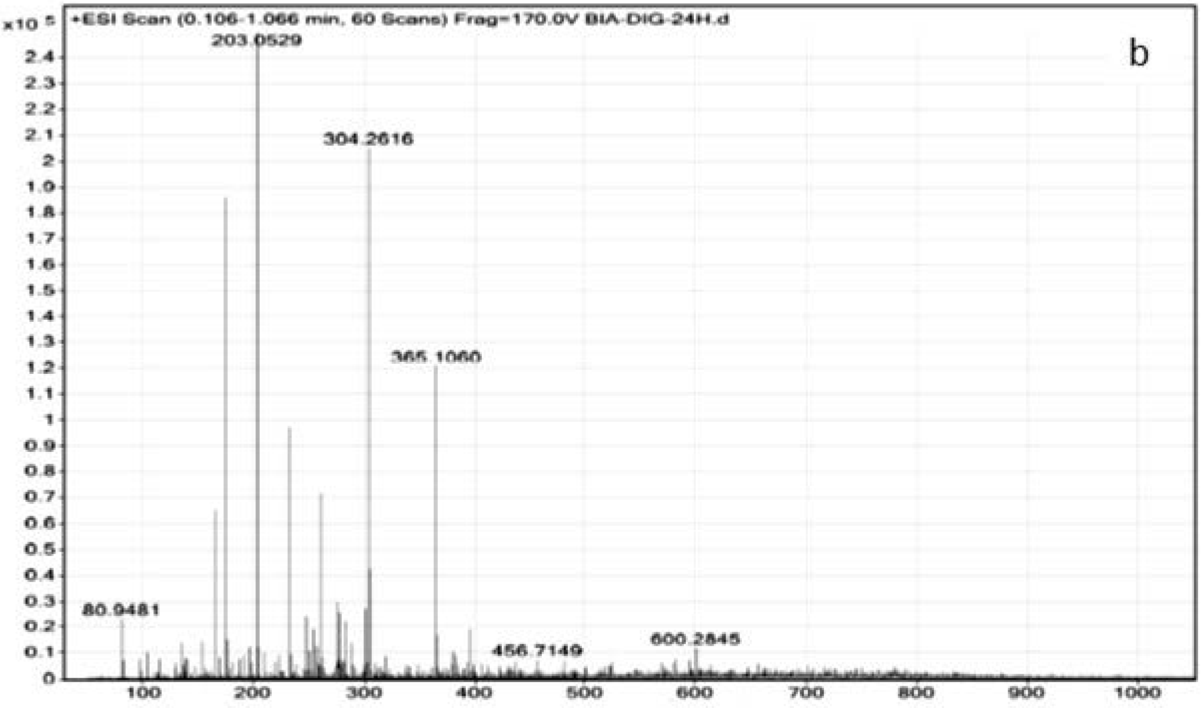
+ESI Scan (0.106-1.066 min, 60 Scans) Frag=170.0V BIA-DIG-24H.d
x10 5
b
203.0529
304.2616
365.1060
80.9481
456.7149
600.2845
